# Tract metastasis from previously unknown malignant pleural mesothelioma: a case report

**DOI:** 10.4076/1757-1626-2-7834

**Published:** 2009-06-29

**Authors:** Tommaso Bartalena, Maria Francesca Rinaldi, Nicola Sverzellati, Vincenzo Allegri, Stefano Fanti

**Affiliations:** 1 Dottorato di Ricerca in Scienze Pneumo-Cardio-Toraciche di Interesse Medico e Chirurgico, University of Bolognavia Belle Arti 42, Bologna, 40126Italy; 2 U.O. di Radiologia III, Pol.S.Orsola-Malpighivia Massarenti 9, Bologna, 40138Italy; 3 Dip. di Scienze Cliniche, Sezione di Radiologia, University of Parmavia Gramsci 14, Parma, 43100Italy; 4 U.O. di Medicina Nucleare, Pol.S.Orsola-Malpighivia Massarenti 9, Bologna, 40138Italy

## Abstract

A case of malignant pleural mesothelioma discovered because of a chest wall metastasis which developed over a previous pleural drainage site is presented. Imaging findings at sonography, contrast enhanced computed tomography and fluorodeoxyglucose - positron emission tomography are shown.

## Case presentation

A 64-year-old Caucasian male from Italy complained of onset of thoracic pain which gradually developed after undergoing a left-sided pleural effusion drainage 9 months earlier; at that time he was dismissed from the hospital without any further investigation since pleural fluid cytologic analysis did not show malignant cells.

Because of progressive worsening of his symptoms he returned to medical attention. Complete cutaneous healing of the drainage insertion site was seen at physical examination which also revealed tenderness of chest wall exacerbated by digitopression.

**Figure 1.**
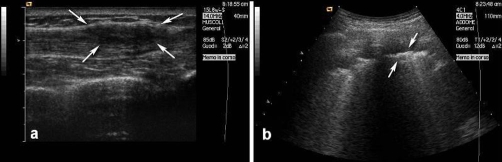
Chest wall ultrasounds showing an ill-defined subcutaneous lesion with inhomogeneous hypoechoic structure **(a)**. Hyperechoic linear thickening of pleural surface consistent with calcified pleural plaques are seen **(b)**.

Ultrasound of the chest wall was performed showing an inhomogeneous hypoechoic subcutaneous soft tissue lesion with ill-defined borders (Figure 1a) corresponding to the prior drainage site. Pleural calcifications and effusion were also seen (Figure 1b) and further imaging was recommended.

**Figure 2.**
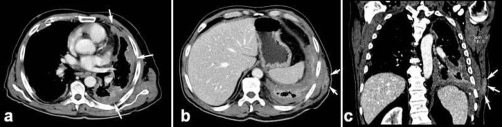
Contrast enhanced CT of the chest showing the pathological circumferential pleural thickening **(a)** and the chest wall tract metastasis **(b, c)**.

A contrast enhanced chest multidetector CT showed left hemithorax volume loss with concentric nodular pleural thickening (Figure 2a) involving the parietal, mediastinal, diaphragmatic pleural surfaces as well as the interlobar fissure. A subcutaneous enhancing soft tissue plaque in the left latissimus dorsi muscle (Figure 2b,c) corresponding to the sonographic finding was also noticed. Thinner scattered partially calcific pleural plaques were seen on the right hemithorax. No mediastinal adenomegalies were present.

**Figure 3.**
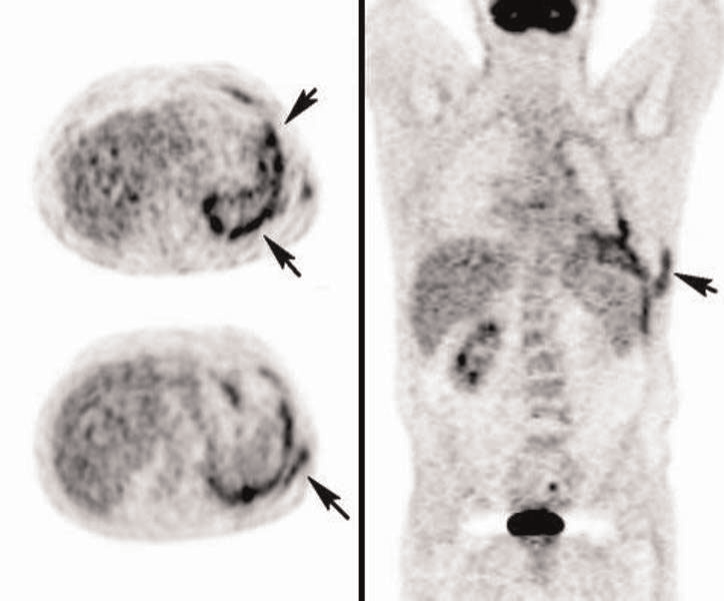
FDG-PET showing striking hypermetobolism in the pleural cavity and chest wall lesions.

An FDG-PET scan was performed showing increased left pleural and chest wall (Figure 3) radiotracer uptake whilst no hypermetobolism was noticed in right pleural plaques. Imaging findings were consistent with drainage tract metastasis from underlying malignant mesothelioma which developed because of professional asbestos exposure (the patient was a former plumber).

CT-guided biopsy subsequently confirmed the diagnostic impressions.

The patient was considered unresectable because of the extensive diaphragmatic involvement and was then referred to an oncologist who treated him with radiotherapy, chemotherapy and palliative care. He passed away 1 year later because of respiratory insufficiency.

## Discussion

Malignant pleural mesothelioma (MPM) is a locally aggressive tumor of the pleura frequently associated to a prior asbestos exposure, which has been estimated to be responsible of about 70-90% of cases [1].

Asbestos induces DNA damage and mutations in mesothelial cells. The carcinogenic effect may be the result of direct effect of asbestos fibers on mitotic spindle of cells disrupting mitosis or be secondary to formation of reactive oxygen species [2].

Other risk factors include previous chest radiation therapy, exposure to Thorotrast contrast medium and SV40 virus infection [3].

The tumor causes progressive nodular pleural thickening that may lead to encasement of the lung in advanced stages. MPM is locally aggressive, with frequent invasion of the chest wall, mediastinum, and diaphragm. Contraction of the affected hemithorax with associated ipsilateral mediastinal shift and hemidiaphragmatic elevation may be seen [4]. However a few cases of contralateral mediastinal shift due to mesothelioma have been also reported in the literature [5]. Distant metastases are uncommon [3].

MPM is associated with a poor median survival, ranging from 4 to 18 months. Treatment is difficult because of the natural resistance of this tumor-entity and single modality therapy gave disappointing results. Multimodality treatment approaches involving surgery with radiation and chemotherapy, seem to be promising in a selected group of MPM patients [3]. Conventional chest radiography usually reveals pleural effusion and/or nodular pleural thickening; however early stages of disease may be overlooked since pleural fluid may hide underlying pleural thickening [4]. CT is superior to radiography for the identification of early abnormalities in patients with MPM and allows better evaluation of the morphology and extent of the pathology [4]. Key CT findings that suggest MPM include unilateral pleural effusion, nodular pleural thickening, and interlobar fissure thickening. Growth typically leads to tumoral encasement of the lung with a rindlike appearance. Calcified pleural plaques are found at CT in approximately 20% of patients [6].

Magnetic resonance imaging can allow improved detection of tumor extension, especially to the chest wall and diaphragm but in the majority of the cases CT and MRI are nearly equivalent and provide similar diagnostic and staging information [7].

FDG-PET is useful in the staging and preoperative evaluation of MPM because of its increased accuracy for detecting mediastinal nodal metastases and its ability to determine the most appropriate biopsy sites [8].

Ultrasounds may show pleural effusions, areas of pleural thickening, calcifications and may be used to assess chest wall invasion with higher sensitivity than CT; drawbacks of sonography are lack of panoramicity and poor acoustic windows in certain regions of the thorax like the subscapular and paraspinal areas [9].

It’s important to remark that pleural abnormalities seen in MPM are not pathognomonic and may be encountered in a variety of conditions like infections (tuberculosis, fungal, actynomicosis), fibrothorax, empyema and metastatic disease to the pleura (most frequently adenocarcinomas arising in the lungs, breast, ovaries and stomach) as well in asbestos-related pleural fibrosis [4].

A tissue diagnosis is therefore required. Unfortunately it is not uncommon to have negative cytology after pleural drainage as it happened in the initial evaluation of our patient; diagnostic yield of pleural fluid cytology may indeed be as low as 20-30% [10].

For this reasons an histological specimen by means of core-biopsies techniques is often necessary to achieve a correct diagnosis [3]. MPM may however complicate these procedures and develop subcutaneous tumor deposits via needle biopsy tracks, surgical scars, and chest tube tracts [11]. It has been estimated that up to 15% of patient with MPM develop chest wall metastases along percutaneous biopsy tracts and drainage canals after aspiration of large effusions. Chest wall metastases rates are even higher (40%) after operative or thoracoscopic excision of biopsy specimens [12,13].

These metastases may cause important pain and discomfort to the patient. For this reason prophylactic drain site radiotherapy in MPM, has been advocated in the past to reduce the incidence of tumour seeding [14] and has been diffusely adopted worldwide; however there’s actually controversy about the effectiveness of this treatment since more recent randomized clinical trials showed no statistically significant difference in occurrence of tract metastases in patients treated with radiotherapy and control groups [15]. Despite these controversies, the possibility of a tract metastases should be kept in mind and warn physicians when a patient with known mesothelioma or previous asbestos exposure develops chest pain over previous pleural intervention sites.

## Abbreviations

CT: computed tomography
FDG-PET: fluorodeoxyglucose - positron emission tomography
MPM: malignant pleural mesothelioma
MRI: magnetic resonance imaging
SV40: simian virus 40
